# Seeking Mind, Body and Spirit Healing—Why Some Men with Prostate Cancer Choose CAM (Complementary and Alternative Medicine) over Conventional Cancer Treatments

**DOI:** 10.4137/imi.s377

**Published:** 2008-02-01

**Authors:** Margaret A. White, Marja J. Verhoef, B.J. Davison, Hal Gunn, Karen Cooke

**Affiliations:** 1 Research Associate, University of Calgary, Calgary, Alberta, Canada; 2 Department of Community Health Sciences, Faculty of Medicine, University of Calgary, Calgary, Alberta, Canada; 3 Department of Urologic Sciences, University of British Columbia, Vancouver, Canada; 4 InspireHealth, Vancouver, Canada; 5 Research Consultant, Vancouver, Canada

**Keywords:** cancer, complementary and alternative medicine, decision-making, quality of life

## Abstract

Little is known about men with prostate cancer who decline conventional treatment and use only complementary and alternative medicine (CAM).

**Objectives:**

To 1) explore why men decline conventional prostate cancer treatment and use CAM 2) understand the role of holistic healing in their care, and 3) document their recommendations for health care providers.

**Methods:**

Semi-structured interviews and follow-up focus groups.

**Sample:**

Twenty-nine men diagnosed with prostate cancer who declined all recommended conventional treatments and used CAM.

**Results:**

Based on strong beliefs about healing, study participants took control by researching the risks of delaying or declining conventional treatment while using CAM as a first option. Most perceived conventional treatment to have a negative impact on quality of life. Participants sought healing in a broader mind, body, spirit context, developing individualized CAM approaches consistent with their beliefs about the causes of cancer. Most made significant lifestyle changes to improve their health. Spirituality was central to healing for one-third of the sample. Participants recommended a larger role for integrated cancer care.

**Conclusion:**

Men who decline conventional prostate cancer treatment and use CAM only may benefit from a whole person approach to care where physicians support them to play an active role in healing while carefully monitoring their disease status.

## Introduction

Complementary and alternative medicine (CAM) use by men with prostate cancer has become increasingly popular in recent years. Studies estimate that 18.2% to 43% [Chan et al. 2005; Eng et al. 2003; Wilkinson et al. 2002; Boon et al. 2003a; Diefenbach et al. 2003; Ponholzer et al. 2003; Hall et al. 2003; Salmenpera, 2002; Lippert et al. 1999] of men use CAM to manage their prostate cancer.

While the majority of cancer patients use CAM in addition to standard care, some choose to forgo conventional cancer treatment and use CAM as an alternative approach. Cassileth et al. (2003) reported that 14% of cancer patients in the United States who used CAM declined conventional treatment; however, their reasons for doing so were not addressed.

A few small qualitative studies with all types of cancer patients have explored reasons for refusing conventional cancer treatment. Results of a small ethnographic study (n = 8) of cancer patients suggest that emotional factors such as anger and fear, sense of control over their illness, and spirituality may influence the decision to decline conventional treatment. These patients also held strong beliefs about the potential of CAM to cure their cancer (Montbriand, 1998). Shumay et al.’s (2001) interviews with fourteen cancer survivors revealed that the desire to avoid damage or harm to the body was the most significant reason for declining conventional treatment. Participants tended to perceive conventional treatment as ineffective and CAM as effective and less harmful. Becoming knowledgeable about CAM contributed to the decision to decline conventional cancer treatment. Huijer and van Leeuwen (2000) interviewed three women with breast or ovarian cancer who refused chemotherapy and three oncologists. Their research revealed that these women’s values, emotions, beliefs and attitudes towards life, suffering and death influenced their decision to forgo conventional treatment. They concluded that what physicians may view as an ‘irrational’ decision, actually resulted from a careful balancing process in the patient’s personal context.

No prevalence data for Canada have been published, however, in previous research we identified 31 cancer patients (including 8 men with prostate cancer) who declined all conventional cancer treatments. (Verhoef and White, 2002). Little is known about which cancer patients make this decision, why they do so, how they are doing over time and what role CAM plays in their cancer management.

Our study explored reasons why cancer patients choose to use CAM in favor of conventional cancer treatment (Verhoef and White, 2002). Potential predisposing factors included a strong belief in whole person healing (versus biomedicine), decision-making preferences, CAM use prior to diagnosis, having a negative experience with mainstream medicine and having a family member die from cancer. Important post-diagnosis factors included doctor-patient communication about treatment options, the emotional impact of the diagnosis, perceived severity of conventional treatment side effects, and increasing sense of control over their illness.

In the secondary data analysis for the eight men with prostate cancer in this study, taking control over all aspects of cancer care, a strong desire to coordinate their complementary and conventional cancer care, and “leaving the door open” to change their decision were important themes (White and Verhoef, 2003). This small secondary analysis also increased our understanding of the clinical and social context, which may explain why some men may be turning to CAM as a first or only option for treating prostate cancer. First, these men were very concerned about the potential adverse effects of prostate surgery (incontinence and sexual functioning), which activated their search for alternative treatments. Second, the growing availability of natural health products, marketed as prostate cancer treatments and the accessibility of information on the Internet made it relatively easy for them to find alternatives to conventional cancer treatment. Third, men with prostate cancer have access to regular PSA blood tests, which provides them with immediate feedback about their disease status. This enables them to assess the risks of their decision, while evaluating the effectiveness of their CAM approach. Prostate cancer is unique in that watchful waiting is sometimes recommended as an option to conventional cancer treatment, particularly for older men. This makes it conceptually challenging to define what constitutes declining conventional cancer treatment for prostate cancer. However, the participants in this study were proactive in seeking alternative approaches to healing, independent of their specialist’s recommendations. The current study is designed to expand on these results and to explore why some men chose CAM instead of conventional cancer treatments, and to understand the role of holistic healing in their cancer care.

In order to expand our knowledge beyond the small sample of prostate cancer patients in the initial study, funding was sought to conduct personal interviews with up to thirty men with prostate cancer who declined all conventional cancer treatment and used CAM as their primary mode of treatment.

## Methods

### Study design and study participants

This is a qualitative study consisting of semi-structured interviews and follow-up focus groups. This paper presents the qualitative component of a mixed methods longitudinal study to assess changes in decision-making and quality of life over three years. Participants were men from British Columbia and Alberta with a confirmed diagnosis of prostate cancer within the past ten years who declined all conventional cancer treatments (surgery, radiation therapy, brachytherapy) recommended by their cancer specialist and were using CAM for their cancer care.

Participants took part in in-person semi-structured interviews at study entry. The interview began by exploring the meaning of the participants’ cancer experience, in relation to their reasons for declining conventional cancer treatments and the use of CAM in their healing approach. In the course of the discussion, the interviewer also elicited information about significant people or sources of information, previous experience with mainstream and holistic medicine, and family history of cancer. Participants were also asked to discuss what made it easier or harder for them to make decisions about conventional and CAM approaches to cancer care.

Focus groups were conducted with participants who took part in the interviews to verify the interview results and to develop patient-centered recommendations for health care providers. The major themes, which emerged in the analysis of the interview data, were presented to participants. Following the presentation of each theme, the facilitator asked two validation questions, “Do these findings accurately represent your experience?” and “Is there anything we have missed that you feel should be included?” recommended for focus group discussions (Krueger, 1994). The focus group also provided the opportunity to explore the connections between themes, in a dynamic interactive group discussion. In the second hour, participants were asked to brainstorm recommendations about how health care providers could best support men with prostate cancer to make decisions about conventional and complementary approaches to cancer care. All ideas were recorded on a flip chart, with the facilitator asking for verification that the recorded items accurately represented their ideas. The discussion was also tape-recorded, transcribed and used to supplement the analysis. Focus group participants also completed a brief questionnaire, indicating how strongly they agreed or disagreed with the health beliefs that emerged as themes in the interviews.

The focus group design incorporated elements of participatory research by creating a forum to involve participants in the data analysis, bring them together around a common health concern, and engage them in the process of developing recommendations for health care providers. (For further discussion of this methodology, see White and Verhoef, 2005)

### Recruitment

A number of strategies were employed to publicize the study in community newspapers, wellness magazines, and regional cancer support groups, as these patients are less likely to be attending a conventional cancer clinic. Health care providers at the Tzu Chi Institute for Complementary and Alternative Medicine and the Centre for Integrated Healing in Vancouver, British Columbia assisted with recruitment. The Clinical Research Ethics Committee of the University of British Columbia and the Conjoint Health Research Ethics Board at the University of Calgary approved the study.

### Data analysis

For both interviews and focus group data, qualitative content analysis was used to analyze the data. This is a systematic approach to organizing and integrating qualitative data according to emerging themes and concepts in order to make valid inferences from the data. Data analysis and data collection were iterative. Audio taped interviews were transcribed and analyzed as the interviews were conducted. Each transcript was read a number of times to get a sense of the depth and meaning of the information, with each researcher (MW, MV) recording thoughts and impressions. After reading several transcripts, a preliminary coding scheme was developed and applied to a sub-set of transcripts to see if new categories and codes emerged, which was revised as needed and applied to subsequent transcripts. This study employed strategies to enhance descriptive validity or accurate accounting of the data and interpretive validity, which refers to verifying the researchers’ interpretation of the data with participants (Sandelowski, 2000). To enhance descriptive validity, the researchers each maintained field notes, documented reasons for coding decisions, and met regularly to compare and combine their independent analysis. To enhance interpretive validity, the focus group discussion and results of the health beliefs questionnaire were used to help verify that the meaning participants attributed to their experience in the interviews was accurately represented by the researchers. The computer software program NVIVO, Version 2.0 (QSR International 2003–2006) was used to assist in the analysis. The focus group data on participants’ responses to the preliminary themes was integrated into the final analysis of the interview data and reporting of the results. Participants’ recommendations are reported on separately.

## Results

### Participants

Sixty-five patients contacted the study coordinator. Twenty-two were excluded because they lived out-of-province, did not meet the medical criteria or were an inappropriate referral. Of the remaining 43 patients, 14 were not interested or did not return the consent form, after receiving the study package. Twenty-nine enrolled in the study for a response rate of 67%. Half of participants were referred by an integrated cancer care center, and about one-quarter responded to a study advertisement in a newspaper or health magazine. Support groups, complementary care practitioners, or other men with prostate cancer referred the remaining participants. See Tables 1 and 2 for a description of participant characteristics and the type of CAM therapies used.

Twenty study participants who took part in the interviews were invited to attend a focus group to discuss the preliminary findings and to make recommendations for health care providers. Five participants did not attend. Reasons for not attending included ‘could not contact’ [2], ‘out-of the country’ [1] and ‘unknown’ [2]. Two focus groups were conducted, consisting of nine and six participants respectively. Each meeting lasted two hours. The two study investigators, (MV and MW), facilitated the focus group.

### Qualitative interviews

The major themes that emerged in the analysis are presented in the context of the cancer journey these participants depicted themselves as being on, as they moved through their cancer diagnosis and early treatment decision-making process, to ultimately create and follow their own healing program.

#### Launching an intensive search for information to evaluate treatment options and seek alternative approaches to healing

Most of the men in this study responded to their initial consultation with cancer specialists by launching an intensive search for information about prostate cancer and treatment options. Their research about conventional treatment tended to reinforce their fears and concerns about long-term side effects:

I had read everything there was and like I said I don’t—why would I go and kill all my good cells in my body and maybe do damage to my organs, that just didn’t make any sense to me.

Some kept abreast of the current research on “watchful waiting” which appeared to increase their confidence that they could safely delay conventional treatment while seeking other options. In the words on one participant:

I found out that there have been no comparative studies on longevity between looking at the two systems. So that kind of set me on my heels … I said ok back off; take your time a bit. So within about 3 weeks I had decided no, I wasn’t going to.

Participants tended to view treatment options in a broader context than conventional medicine. They empowered themselves to explore the risks and benefits of conventional treatment and also put considerable effort into researching CAM approaches to heal from prostate cancer. Based on what they learned from books, Internet sites, natural health care practitioners and physicians knowledgeable about CAM, they designed their own healing approach. Some used a specific book such as “Prostate Health in 90 Days Without Drugs or Surgery” by Larry Clapp (1997), as a basis for their healing program.

I know exactly where I’m going and it was easy right from the start. Right from the beginning, Larry Clapp’s book is where I’m going. That was the route I was going. I was firmly convinced of that … it was very inspirational. And now that I have read hundreds of books since then and they just confirm what I’m doing.

#### Learning through the “lived experience” of others who received cancer treatment

Experiential knowledge derived from knowing others treated for cancer seemed to have a powerful effect on shaping their perceptions about conventional cancer treatment. Many participants contacted men with prostate cancer as part of their search for treatment information. This experience tended to reinforce their fears and concerns about conventional treatment:

I went to the support group and started talking to all kinds of guys who had all kinds of treatment and what struck me were the dramatic side effects and the dramatic recurrence. In fact, it really struck me.

Overall, participants perceived men treated for prostate cancer as having experienced a significant loss of social identity (depression, social isolation, change in personality), and loss of sexual identity. They tended to perceive men who received conventional treatment as victims, while viewing those who refused treatment as more optimistic. This raised many quality of life concerns and reinforced their desire to seek natural healing approaches.

The interviews also revealed how past experiences with loved ones who died from cancer may influence current treatment decisions. Several participants had lost one or more family members to cancer. In the interviews, they described the loss of quality of life their family member experienced while undergoing cancer treatment. This experience lessened their faith in the effectiveness of conventional medicine and influenced them to focus on quality of life when making their own treatment decisions.

Well yeah she [his wife] died of cancer, respiratory cancer at that time so I decided that’s not the way I want to go. So I didn’t want to have certain medical things because it seemed that they just keep taking parts of you and finally they say there is nothing left to take, we can’t help you any more.

#### Control over treatment decision-making and healing approach

Taking control over treatment decision-making, cancer management and their healing approach emerged as an important theme in the interviews. At the outset, participants took control by delaying conventional treatment, so they could try alternative healing approaches first. As described earlier, they took control by searching for information to evaluate conventional and complementary approaches to healing from cancer. Overall these men took a high degree of responsibility for their health, and were committed to making many changes in their lives to bring about healing. They wanted physicians who could support them to take an active role in their healing, and put a great deal of effort into developing a collaborative relationship with their physicians. Most were having regular PSA tests, which they used to evaluate their disease status and the effectiveness of the complementary therapies they were using. They fine-tuned their healing program as they went along, depending on the results.

The theme on taking control generated considerable discussion in the focus group about how taking personal responsibility for one’s health and the consequences of one’s decisions relates to healing. For some, taking control brought on good feelings of empowerment, and an overall improvement in their health. In the words of one participant—“*It’s healthful. It feels good to feel you are in control. It brings on a feeling of well being.”* One person felt control was a consequence of the decision to decline conventional cancer treatment, rather than something he wanted. Another saw taking control as necessary for self-preservation—he knew himself better than anyone else and needed to take responsibility for his own survival. In the course of the discussion, the distinction was made that healing related to those things you have control over *“what you do (healing, lifestyle changes, internal changes, taking responsibility for one’s health, immune system)* while curing related to those things you have no control over *“(cure, diagnosis of cancer, doctor doesn’t give you control over information)”.*

#### Seeking a healing approach consistent with beliefs about causes of cancer

Health beliefs played an important role in the decision to use CAM as a first option to treat prostate cancer. Participants expressed the belief that Western medicine tended to focus on treating the tumor while downplaying the impact of treatment on their emotional, social and spiritual well being. Some participants felt that the focus on curing (versus healing) caused some physicians to rush patients into treatment decisions, thus intensifying fear and anxiety. As the men in this study tended to focus on “healing”, they sought a treatment approach that addressed the interrelationship between the mind, body and spirit. One participant used the metaphor of a pie to explain his view of a healing approach:

For me it’s important it’s a lot of emotions, a lot of different bad thinking. … We have to heal that too … —the way I look at it it’s like a big pie. Dealing with the physical is one slice of the pie and I don’t believe in that. I believe that if people want to heal … allopathic medicine is one piece of the pie, meditation is one piece of the pie, conscious breathing is one piece of the pie, and visualization would be another piece of the pie. Then working on emotions.

Participants also expressed the belief that for a treatment to be effective, it needs to address the underlying cause(s) of cancer. They had many ideas about what causes cancer, including environmental conditions (radiation, toxins) lifestyle (stress, poor diet, harmful lifestyle behaviors), and medical interventions, including cancer treatments. Concerns were expressed that radiation therapy would cause a recurrence or damage internal organs, and that biopsies or surgery could damage the prostate, possibly causing cancer cells to spread. Another concern was that cancer treatment weakens the immune system, making it difficult for the body to heal. Finally, many participants expressed the view that you have to believe in the treatment for it to work, whether taking a conventional or complementary approach.

Participants sought approaches to healing their cancer that were consistent with their beliefs about causes of cancer. There were four principles that guided their CAM approaches: 1) creating the conditions in the body so that the cancer cannot thrive 2) strengthening the immune system through a healthy lifestyle 3) activating the mind or inner resources to aid in the healing of cancer and 4) eliminating major sources of stress thought to have contributed to their cancer. Many expressed the belief that unless one addressed these underlying conditions, conventional treatment would not be successful in eliminating their cancer:

About two weeks before the surgery, I decided not to do the surgery. Dr. X is one of the best surgeons around but I still felt that it wasn’t the right way for me to go. I wasn’t going to—even if the surgery got everything out, I felt that from what I had learned, I felt my environment in my body the cancer was going to come back. I had not changed my life enough and it was not going to make a difference.

The focus group discussion revealed the connections between sense of control and health beliefs. As one participant put it *“taking control means living your belief system”*. The discussion also revealed the belief that cancer can only be resolved by taking control over one’s health—*“the cause and cure of cancer is based in control, once you learn how to balance control, the cancer will resolve itself”.*

Focus group participants also completed a questionnaire asking them to indicate how strongly they agreed or disagreed with the health beliefs which were identified in the interviews. The results suggest there is a high consensus (more than 70% “strongly agreed”) among participants in the beliefs that 1) Western medicine treats the tumour not the whole person, 2) Conventional cancer treatment does not address the causes of cancer, weakens the immune system and can damage the internal organs making it difficult to recover from cancer 3) Holistic treatment strengthens the immune system and 4) You have to believe in the treatment for it to work. There was a low consensus (Less than 30% “strongly agreed”) that Western medicine excels at diagnosis of illness. [See Table 3]

#### Cancer as part of a spiritual journey

Spirituality played a central role in decision-making and cancer recovery for some participants (White and Verhoef, 2006). Half of the participants used mind-body therapies and one-third also used spiritual practices as part of their cancer care [Table 2]. Some men viewed their cancer as part of a spiritual journey and searched for meaning in their cancer experience. They viewed cancer as a “gift” that had bought many blessings into their life, or a “teacher” that had deepened their insights into the meaning of life. One participant described how his spirituality transformed his cancer diagnosis into a positive experience:

I really, really had a very, very strong sort of intuitive sense that this illness is … a spiritual journey and it has been incredibly wonderful actually. I almost remember the first day of diagnosis; I could never describe it as anything else but a gift.

Some participants expressed concern about how surgery or other treatments might interfere with their spiritual practice, which was important to their healing. Some men drew on their spiritual practice and faith in a divine presence to guide their treatment decision-making. A few men engaged in an intensive process to heal their prostate cancer, using spiritual practices.

A diagnosis of a life-threatening illness in these men seems to encourage an exploration of authenticity and connection to meaning. Having a cancer diagnosis also appears to have influenced their spirituality by deepening spiritual practice, strengthening links with spiritual community and improving relationships. (See White and Verhoef, 2006 for elaboration of this theme.)

#### Healing outcomes

Being diagnosed with prostate cancer served as a catalyst for many of these men to undergo a significant transformation in their lifestyle, with the belief that addressing the imbalances in their life would activate the body’s natural healing processes.

You have got to change your lifestyle. You have got to change the way you think. You have got to change the way you eat. You have got to start exercising. I meditate, I exercise, I eat fruits and vegetables and I pray. I do everything I can possibly do to help myself and its definitely paying off because I feel so utterly fantastic.

While it is not in the scope of a qualitative study to evaluate outcomes, the interview data do provide some insights into the healing outcomes participants identified as important to them. These included enhanced physical well being (e.g. increased energy, resolution of chronic health conditions), a heightened sense of emotional well-being, an increased appreciation of the meaning of life, and enriched social relationships.

So it has been a stimulus I think for sort of a much more meaningful, not meaningful, it has gone beyond the—some of the literature at the Centre says that cancer can be a gift in opening up whole new horizons and it has been I think that for us and our family in that probably out of this is coming richer relationships, richer health as a result of the stimulus of this whole process and its all focused around complementary approaches.

Some participants expressed a cautious awareness that feeling good and having no symptoms did not necessarily mean their cancer was gone.

#### Support during the healing journey

In the course of the interviews, participants identified sources of support that were integral to their healing journey. Those who were in a personal relationship expressed their appreciation for how deeply their wives or partners shared and supported them in their decision-making and healing process. Conflict over their decision was more likely to arise with adult children. Participants tended to seek out physicians who could support them in their health beliefs. Many participants reported how the support of their family doctor was central to their healing. Cancer specialists tended to be less involved after the initial consultation, although one participant described how integral the support of his cancer specialist was to his recovery.

About half of the participants attended the Centre for Integrated Healing and described the benefits they experienced from attending an integrated cancer care program. These participants valued having a medical doctor who encouraged them to play an active role in their healing, assisted them to evaluate the safety and efficacy of CAM therapies, and provided ongoing monitoring and evaluation of their cancer. Other benefits reported included having a framework in which to develop their own healing approach, feeling part of a team, and learning strategies to reduce stress and make lifestyle changes.

### Participants’ recommendations

In the second part of the focus group, participants were asked to make recommendations for how health care providers can best support men with prostate cancer to make decisions about conventional and complementary approaches to cancer care. Five major recommendations emerged out of the discussion, most directed towards physicians. First, they recommended that physicians encourage men to take the time they need to adjust to a cancer diagnosis and make a treatment decision. Not doing so may push men away from the cancer system:

“When people go into shock—make it clear to people there is time to investigate options, and don’t rush people into surgery.”

Second, they would like physicians to consider how cancer treatment affects all aspects of their well being, rather than focusing only on the physical aspects of their cancer:

Listen to me as a whole person. Not just this one little bit. It’s a problem, cut it out. There is a whole man here and a whole bunch of things in life that we can’t see. Take care of this whole person.

Third, they recommended that doctors encourage and support men to play an active role in their health decisions and recovery from cancer:

I think that doctors should encourage patients to be proactive and look at other solutions and often times they don’t.

Fourth, they recommended that physicians be open to assisting men to find a physician who can support them in their philosophy of healing:

Dr. S. talks about looking for a physician as a true healer and I think that doctors should remember that it’s really what the patient wants. He wants to find someone who identifies with him, who really wants to work, as a team with him and the doctor should encourage the patient to leave if either of them is not comfortable. I think they should be open to that.

In particular, participants wanted family doctors to be aware of and refer patients to integrative cancer care services, where available:

I would like to see … my GP… or whoever has at his or her disposal information that they could give to me where they would likely send me to the Centre for Integrated Healing. Send me to a place that ...

Fifth, participants recommended that the government increase support for integrative cancer care by funding integrated medicine clinics, including some CAM therapies under Medicare, and removing barriers that make it difficult for physicians to integrate CAM into their medical practice.

## Discussion

This study confirmed our earlier research that both a sense of control and health beliefs play an important role in the decision to decline conventional treatment and to seek a more holistic approach to healing. The research also provided an in-depth understanding about how spirituality, and beliefs about causes of cancer shaped treatment decisions. These men were seeking healing in a broader context of the mind, body and spirit by taking control over their cancer care and seeking physicians who could support them to play an active role in their own healing. Most made significant changes in their lifestyle and many had intensified the use of mind-body and spiritual practices. Underlying this approach was the belief that the cancer would return if they did not address the emotional, spiritual and lifestyle factors that may have contributed to their cancer. They emphasized quality of life, and were concerned about how conventional treatment may impact on all aspects of their well-being. At the same time, they valued the medical expertise of conventional doctors and sought out physicians who could support them in their beliefs while carefully monitoring their disease status.

There are some limitations to this study. It is a small sample and likely under-represents men who are not doing well, or may have died. Many of these men attended a structured integrative cancer care program, which may have influenced the content of the interview data. Nor can this qualitative study tell us how their decision to use an alternative approach in favor of conventional cancer treatment affects their disease status or quality of life over the long-term. The results of the longitudinal study will report on the changes over three years in their treatment decisions, how they feel about their decision, quality of life and disease status.

## Clinical Recommendations

The considerable commitment and energy these men devoted to improving their health, and the confidence they expressed in managing their cancer care were remarkable findings. While there are potential risks associated with declining conventional cancer treatment, our research suggests there are also positive psychological and spiritual outcomes associated with the transformative changes they made as a result of empowering themselves to take control over their treatment path. Several clinical recommendations arise from these findings. First, it is important to be aware of the role of health beliefs in the formation of treatment choices. This study suggests that beliefs about the underlying causes of cancer shape the decision to decline conventional cancer treatment and inform their approach to holistic cancer care. Huijer and van Leeuwen’s (2000) research found a considerable gap between how oncologists and patients viewed the decision to decline conventional cancer treatment, suggesting that patients were seeking to balance personal values, emotions, and beliefs. There is a need to link individuals who delay or decline conventional treatment to integrative cancer care services, both to support them in their healing approach and to ensure they are receiving adequate follow-up medical care. As integrative cancer care is based on a model of whole person healing, this approach provides a context for individuals to consult with physicians about how to best achieve a personal balance in emotional, spiritual and physical dimensions of healing.

Second, health care providers can reassure these men that the spiritual resources they have developed will also be of benefit should they decide to have conventional cancer treatment. For example, Krupski et al.’s study (2005) reported that higher levels of spirituality is associated with improved quality of life outcomes for men who undergo conventional treatment for prostate cancer compared to those with low spirituality. Some of the participants in our study worked well with metaphor and imagery, which has also been shown to be beneficial in helping patients to cope with conventional treatment and its side-effects (Rossman, 2002).

Third, our research suggests that learning through the ‘lived experience of others’ who have experienced cancer treatment may play a powerful role in shaping perceptions about conventional cancer treatment. It is important for health care providers to explore how past experiences with loved ones who died from cancer may be influencing current decisions about treatment. Some patients may have unresolved issues about the loss of a loved one or the treatment and care experienced by their family member. They may also have incomplete information about how their type and stage of cancer compares to that of their family member.

Finally, these men were committed to transforming their lifestyle in an effort to maintain their quality of life and slow down the progression of their disease. Current research suggests that lifestyle improvements such as diet and exercise (Kroenke et al. 2005; Holmes et al. 2005; Ornish et al. 2005) or mind-body healing programs (Cunningham 2005a, 2005b) may result in improved disease outcomes for some cancer patients. Ornish et al. (2005) randomized 93 men with prostate cancer on watchful waiting to a comprehensive lifestyle program or a usual care control group. They reported that none of the men in the intensive one-year lifestyle program experienced disease progression while six of those on watchful waiting only went on to conventional treatment due to an increase in PSA and/or progression of disease on magnetic resonance imaging. These studies suggest there are actions men with prostate cancer can take to improve both their quality of life and disease outcomes. Klotz (2005) and Klotz and Nam (2006) suggest that some men with early stage prostate cancer may be over treated and could safely delay having conventional treatment if monitored under an active surveillance program. Men with prostate cancer who are seeking whole person healing, would greatly benefit from health care providers who can acknowledge and support their commitment to transforming their health while carefully monitoring their disease status and assessing the risks associated with declining conventional treatment.

## Figures and Tables

**Table 1.** Characteristics of participants (N = 29).

**Characteristic**	**Category**	**#**	**% Total**
Age	50–59 yrs	7	(24.1)
	60–69 yrs	8	(27.6)
	≥ 70 yrs	14	(48.3)
	Mean = 67.5		
	Range (50, 85)		
Martial status	Married	23	(79.3)
	Single/No longer married	6	(20.7)
Education level	High school or less	6	(20.7)
	Technical or some university	11	(37.9)
	University degree or higher	12	(41.4)
Employment status	Employed/self-employed	14	(48.3)
	Retired	14	(48.3)
	Unemployed	1	(3.4)
Time since diagnosis at study entry	<1 year	8	(27.6)
	>1 and <5 years	15	(51.7)
	5 to 10 years	6	(20.7)
	Mean = 34.2 months		
	Range (2 months, 10 years)		
Conventional treatment declined *(Based on patient’s understanding of the treatment(s) his cancer specialist recommended)*	Surgery	21	(72.4)
	Radiation therapy	12	(41.4)
	Brachytherapy	9	(31.0)

**Table 2.** Type of CAM therapies used for prostate cancer (n = 29).

**CAM therapy**	**# patients**	**% patients**
Vitamins and supplements	27	93.1
Herbal Supplements	26	89.7
Diet/Body therapies	26	89.7
Body/energy therapies	18	62.1
Body/mind therapies	16	55.2
Body/faith healing	11	37.9
Physical manipulation	11	37.9
Extracts and concentrates	8	27.6
Chemicals and synthetics	3	10.3

**Table 3.** Health beliefs (Based on health beliefs expressed by participants in the interview research) n = 15 focus group participants.

	**Strongly disagree**	**Disagree**	**No opinion**	**Somewhat agree**	**Strongly agree**	**No response**
**Beliefs expressed that Western medicine**						
Is controlled by economic interests		1 (7%)		9 (60%)	5 (33%)	
Excels at repairing the body	1 (7%)	1 (7%)		7 (47%)	6 (40%)	
Excels at diagnosis	2 (13%)	1 (7%)	1 (7%)	7 (47%)	4 (27%)	
Factory-like	1 (7%)	1 (7%)		6 (40%)	7 (47%)	
Military model		1 (7%)		6 (40%)	6 (40%)	4 (27%)
Treats tumor not the person	2 (13%)			3 (20%)	10 (67%)	
Ignores spirituality				8 (53%)	7 (47%)	
**Beliefs expressed about causes of cancer**						
Environmental conditions			1 (7%)	6 (40%)	7 (47%)	1 (7%)
Poor lifestyle habits			1 (7%)	6 (40%)	7 (47%)	1 (7%)
Cancer treatments		1 (7%)	2 (13%)	4 (27%)	6 (40%)	2 (13%)
**Beliefs expressed about cancer treatment**						
Does not address causes of cancer				4 (27%)	10 (67%)	1 (7%)
Surgery can damage the prostate, causing cancer cells to spread				4 (27%)	9 (60%)	2 (13%)
Radiation therapy can cause a recurrence			1 (7%)	4 (27%)	7 (47%)	3 (20%)
Weakens the immune system				3 (20%)	11 (73%)	1 (7%)
Damages internal organs, making it difficult to recover from cancer				2 (13%)	12 (80%)	1 (7%)
**Beliefs expressed about holistic approaches to cancer care**						
Addresses underlying causes of cancer				4 (27%)	9 (60%)	2 (13%)
Creates conditions so the cancer cannot thrive				5 (33%)	8 (53%)	2 (13%)
Strengthens immune system				2 (13%)	11 (73%)	2 (13%)
Mind-body practice promotes healing of cancer				4 (27%)	8 (53%)	3 (20%)
You have to believe in the treatment for it to work.				3 (20%)	10 (67%)	2 (13%)
